# Prediction of Tattoo Removal Sessions With Picosecond Lasers: Development and Validation of a Novel Clinical Scale With Machine Learning Techniques

**DOI:** 10.1111/jocd.71088

**Published:** 2026-07-27

**Authors:** Valerio Pedrelli, Emanuele Tauro, Matteo Tretti Clementoni, Alessandra Zevini, Riccardo Barini, Enrico Gianluca Caiani

**Affiliations:** ^1^ Laserplast SrL Milan Italy; ^2^ Dipartimento di Elettronica Informazione e Bioingegneria, Politecnico di Milano Milan Italy; ^3^ IRCCS Istituto Auxologico Italiano, San Luca Hospital Milan Italy; ^4^ El.En. Group Calenzano Italy

**Keywords:** machine learning, picosecond laser technology, predictive scale, tattoo characteristics, tattoo removal

## Abstract

**Background:**

Accurate prediction of the number of sessions required for laser tattoo removal is essential for effective treatment planning and patient counseling. The Kirby–Desai (KD) scale, developed for Q‐switched nanosecond lasers, overestimates treatment sessions when applied to modern picosecond laser technology.

**Aims:**

To develop and validate a novel predictive scoring system specifically calibrated for picosecond laser tattoo removal and to compare its predictive accuracy with the KD scale.

**Methods:**

A retrospective cohort of 545 patients treated with picosecond laser tattoo removal between 2019 and 2024 was analyzed. Tattoo characteristics were evaluated using both the KD scale and the newly developed (PT) scale, which incorporates six parameters: tattoo type, pigment density, anatomical location, ink color, scarring, and layering. Multiple regression and machine learning models were trained separately on KD‐ and PTbased variables. Model performance was assessed using mean absolute error (MAE), root mean square error (RMSE), and coefficient of determination (*R* ^2^) on an independent test set. Model interpretability was evaluated using SHapley Additive exPlanations (SHAP).

**Results:**

PT‐based models consistently outperformed KD–based models. The PT Ridge regression model achieved the best predictive performance (MAE = 0.58, RMSE = 0.64, *R* ^2^ = 0.73), significantly improving accuracy compared with KD‐based approaches (*p* < 0.001). SHAP analysis identified tattoo type and pigment density as the most influential predictors.

**Conclusions:**

The PT scale provides a more accurate and clinically relevant prediction of treatment sessions for picosecond laser tattoo removal than the KD scale, offering a practical and reliable tool for contemporary clinical practice.

## 1 Introduction

The foundation of modern laser tattoo removal rests upon the principle of selective photothermolysis, as proposed by Anderson and Parrish [1]. This theory established two fundamental requirements for selective destruction of a target: first, the laser energy must operate at a wavelength highly absorbed by the target relative to surrounding normal tissue; second, the pulse duration must be shorter than the thermal relaxation time (TRT) of the target, preventing heat dissipation through conduction to adjacent tissues. These principles enabled the development of Q‐switched nanosecond lasers, which revolutionized the treatment of pigmented lesions and tattoos.

Q‐switched lasers deliver highly concentrated energy pulses that generate extremely precise temperature elevations within the specific targets, effectively minimizing thermal dissipation to peripheral tissues. This interaction produces plasma formation resulting in physical fragmentation of tattoo pigments which are subsequently cleared via lymphatic drainage. This selective targeting minimizes photothermal complications, establishing Q‐switched lasers as the gold standard for tattoo removal for over two decades [2].

Clinical experience revealed significant heterogeneity in treatment response. Not all patients respond equally to Q‐switched laser tattoo removal, highlighting the critical importance of pre‐treatment analysis of multiple variables. Individual skin characteristics and specific tattoo characteristics including ink density, color type, and anatomical location all profoundly influence treatment parameters and outcomes [3].

To address this variability, the Kirby‐Desai (KD) scoring system was introduced in 2009 as a standardized tool for predicting treatment outcomes [4]. Based on retrospective analysis of Q‐switched laser treatments, this scoring system quantifies six critical parameters: skin type, anatomical location, ink color and amount, scarring or tissue change, and layering. Each parameter is assigned a numerical value, with the total score correlating to the estimated number of sessions required for complete clearance, enabling more accurate patient counseling and treatment planning [4].

The potential of pulse widths shorter than the TRT of tattoo particles was first demonstrated in the late 1990s through experimental picosecond laboratory systems [5]. Despite these promising early results, commercial implementation remained unavailable for over 15 years; the paradigm shift occurred in 2012 with FDA approval of the first commercially available picosecond laser for tattoo removal. Subsequent clinical investigations have consistently demonstrated superior efficacy compared to Q‐switched nanosecond systems, with enhanced pigment fragmentation and reduced collateral thermal damage [6, 7, 8, 9]. These technological advances translate into tangible clinical benefits, particularly as enhanced safety profiles and significantly reduced treatment sessions for complete clearance.

This evolution has diminished the predictive accuracy of the Kirby‐Desai scale. Originally calibrated exclusively for Q‐switched nanosecond laser technology, which typically requires 10–15 sessions for the complete removal of professional multicolor tattoos, its application to modern picosecond protocols leads to systematic overestimation of the required treatment course. This divergence is significant, as clinical evidence shows that 30%–50% fewer sessions are actually needed with picosecond systems, which commonly achieve clearance in only 5–8 sessions [10, 11, 12].

Accurate prediction of the number of treatment sessions required for tattoo removal is fundamental to effective clinical practice and patient satisfaction. During pre‐treatment consultation, patients require realistic expectations regarding treatment duration, total cost, and commitment required to achieve their desired outcome. Overestimation of treatment sessions can deter patients from initiating therapy or lead to unnecessary financial burden, while underestimation may result in frustration, premature treatment discontinuation, and negative perceptions of treatment efficacy. In an era where patient‐centered care and shared decision‐making are paramount, clinicians require evidence‐based predictive tools that accurately reflect contemporary laser technology and treatment protocols.

This study addresses the critical need for an accurate, validated scoring system specifically calibrated to picosecond laser tattoo removal. Through a retrospective evaluation of a large and heterogeneous patient cohort, our primary objective was to develop and validate a novel scale together with a comprehensive predictive machine learning model that demonstrates superior accuracy compared to the Kirby‐Desai score when applied to patients treated exclusively with picosecond laser technology. We aimed to achieve this by incorporating some traditional parameters alongside previously unquantified variables, including a refined classification of tattoo types. Ultimately, by providing clinicians with a reliable, evidence‐based tool for treatment planning, this model seeks to enhance patient counseling, optimize treatment expectations, and thereby improve overall satisfaction with picosecond laser tattoo removal outcomes.

## 2 Materials and Methods

### 2.1 PT Scale Description

Tattoo characteristics and treatment complexity were systematically evaluated using the PT scale, a novel predictive tool specifically developed and validated for picosecond laser technology. This scale quantifies six key parameters that influence the number of treatment sessions required for complete tattoo clearance: tattoo type, pigment density, anatomical location, ink color, presence of scarring, and layering (cover‐up tattoos). Complete tattoo clearance was defined as the absence of visible residual pigment within the previously tattooed area, as assessed by direct clinical examination. Outcome assessment was performed by the clinician who had not administered the treatment in each case, ensuring separation between treating and evaluating physicians throughout the dataset.

Each parameter is assigned a numerical score based on its anticipated impact on treatment difficulty (see Table 1 for detailed scoring criteria). Tattoo type is categorized according to artistic style and technical complexity: tribal tattoos (7 points), lettering/script (5 points), old school/blackwork (4 points), geometric or ornamental designs (3 points), and realistic/etching (2 points). Additional classification distinguishes professional from amateur application: amateur tattoos receive a 2‐point deduction from the total score, reflecting the typically lower pigment density and less uniform ink deposition associated with non‐professional technique.

**TABLE 1:** PT scale scoring system. For each parameter different options are presented, together with the corresponding points to be assigned.

Parameter	Options		Points
Tattoo Type	Tribal	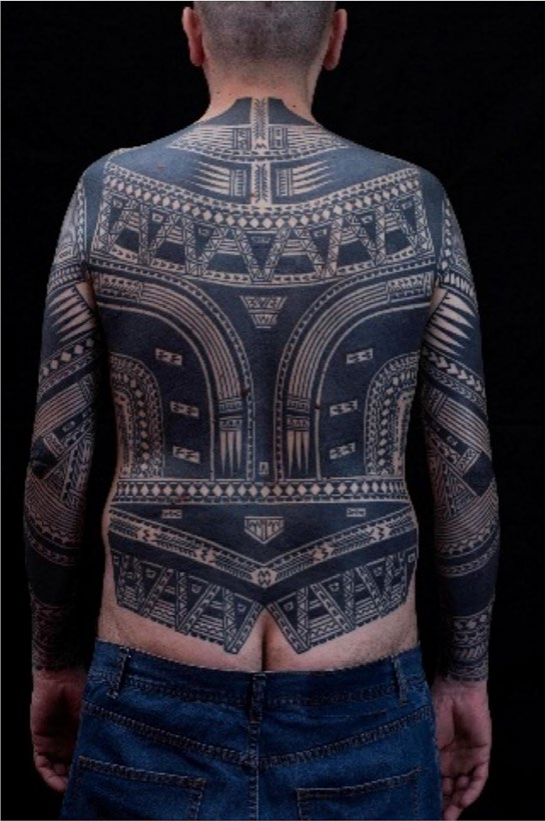 *Image provided by* Marcowallacetattoo, *with permission*.	7
	Lettering/Script	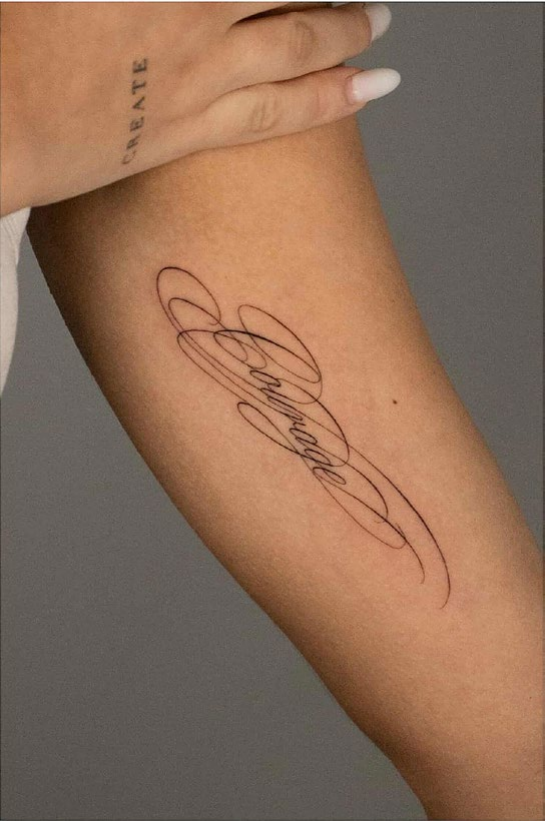 *Image provided by* _asiasharp_, *with permission*	5
	Old School/Blackwork	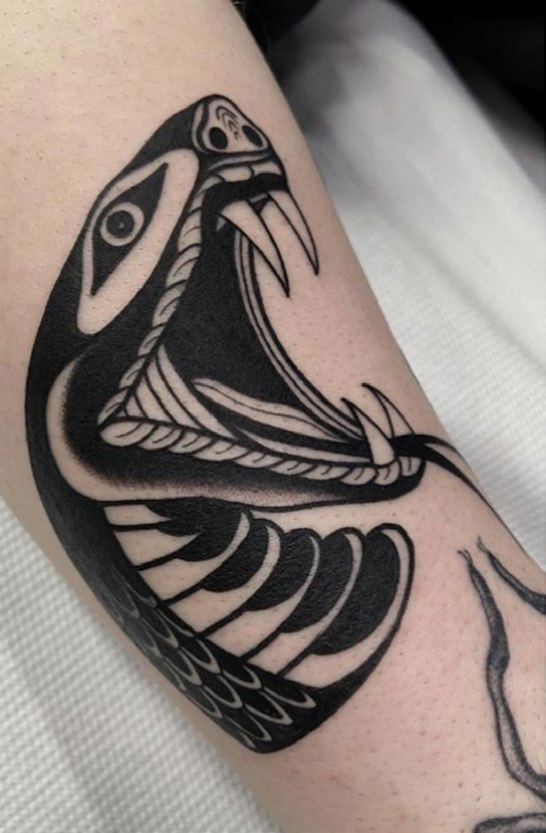 *Image provided by* stefaniapallestrinitattoist, *with permission*	4
	Geometric Or Ornamental	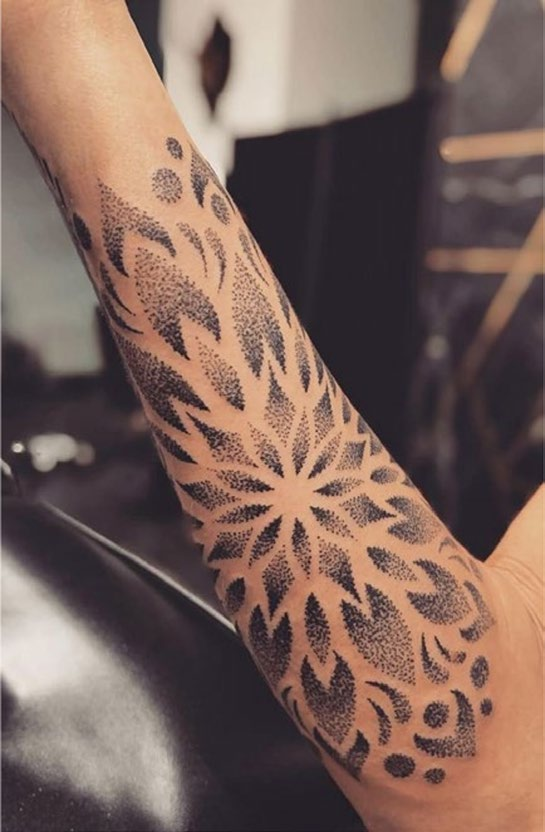 *Image provided by* norinatattoo, *with permission*	3
	Realistic	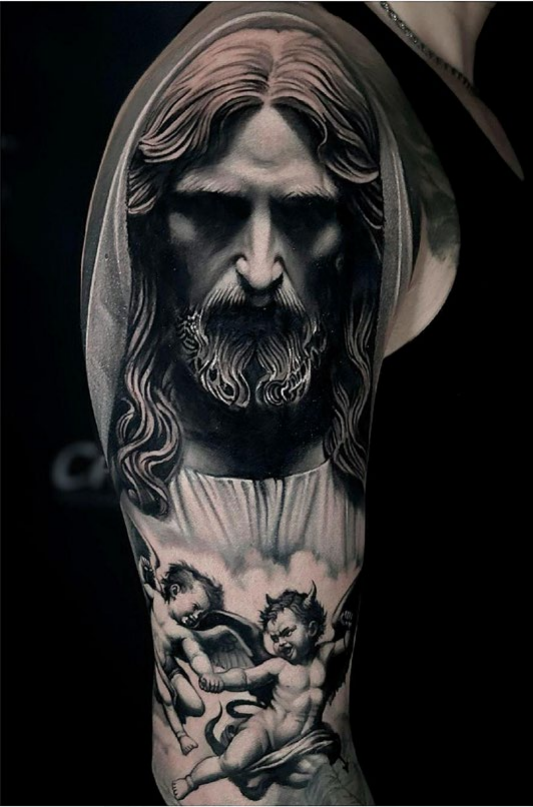 *Image provided by* giuseppe_gibi_bonelli, *with permission*	2
	Etching	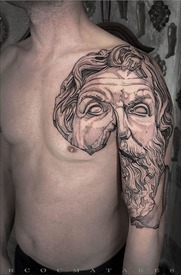 *Image provided by* marcomatarese, *with permission*	2
Pigment Density	High	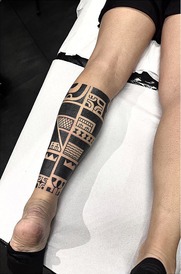 *Image provided by* luigimarchinitattoos, *with permission*	3
	Medium	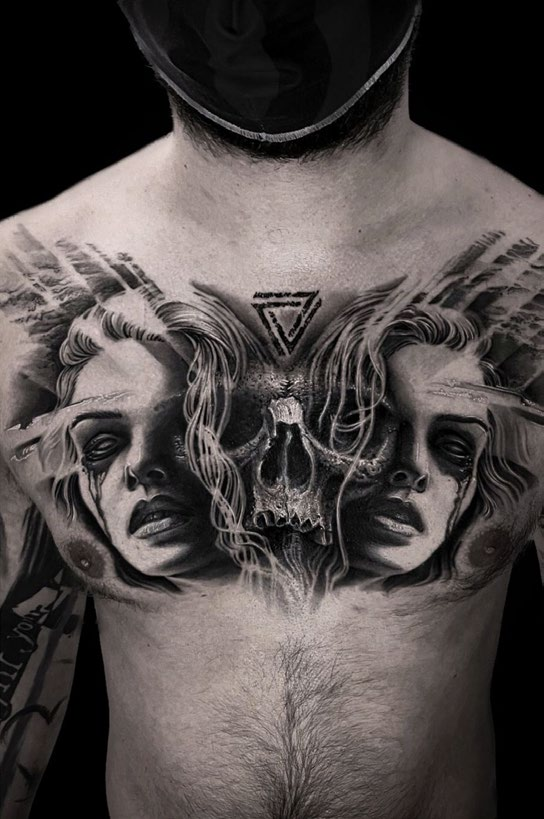 *Image provided by* clod_the_ripper_tattoo, *with permission*	2
	Low	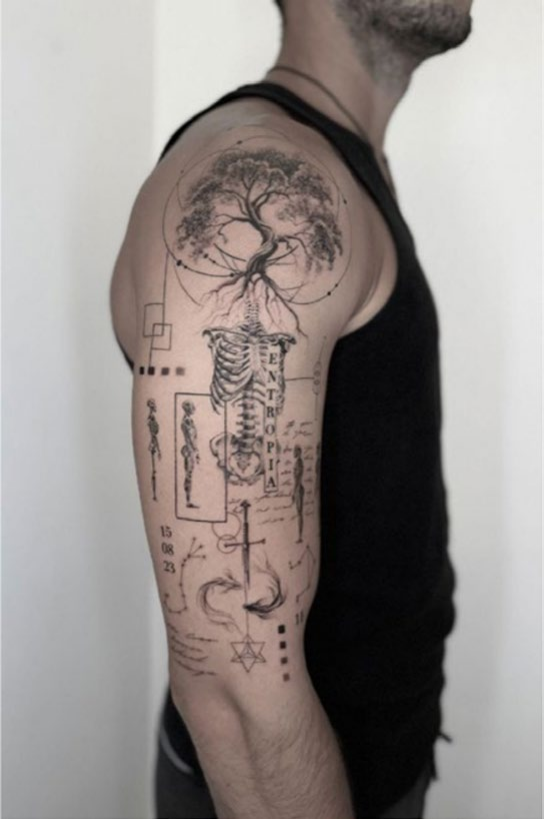 *Image provided by* ellyson_restelli, *with permission*	1
Anatomical Location	Hands/Feet/Bony prominences	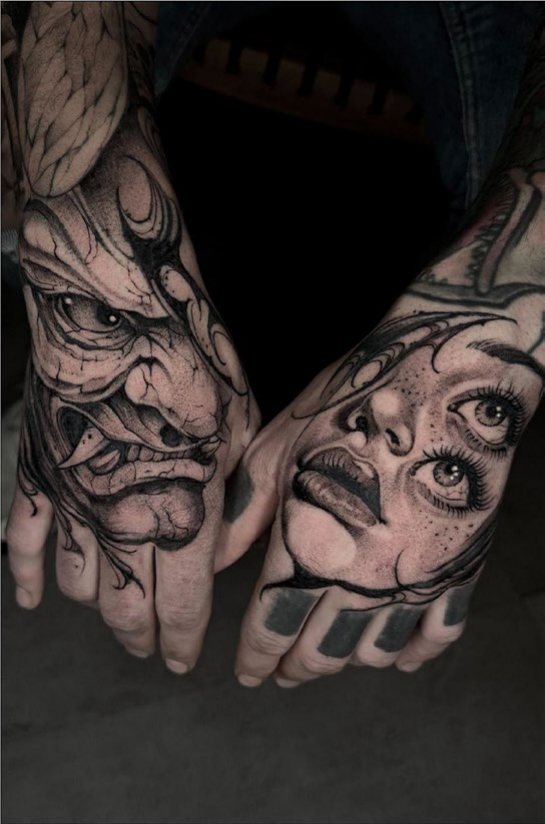 *Image provided by* fra_koto, *with permission*	2
	Rest of body	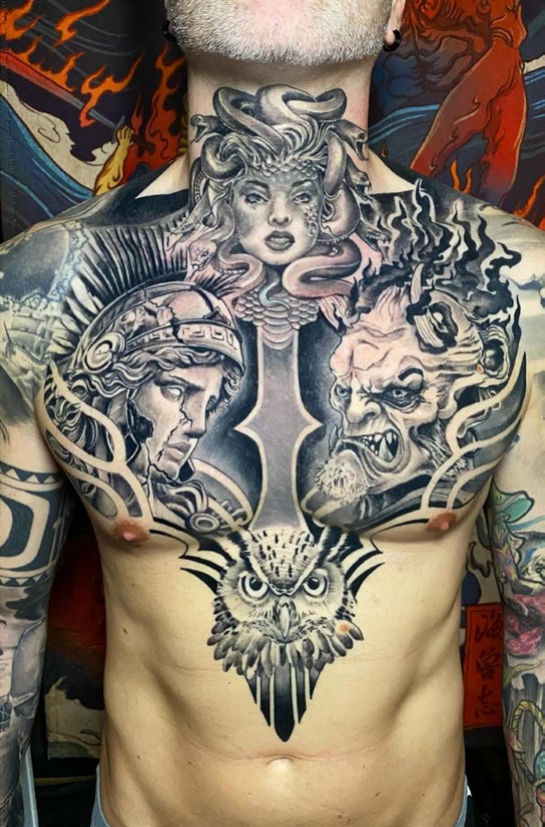 *Image provided by* davide_dak_controguerr, *with permission*	1
Ink color	Black or Red only	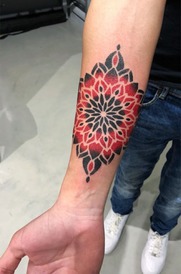 *Image provided by* loscoboy, *with permission*	1
	Multicolor (no pastels)	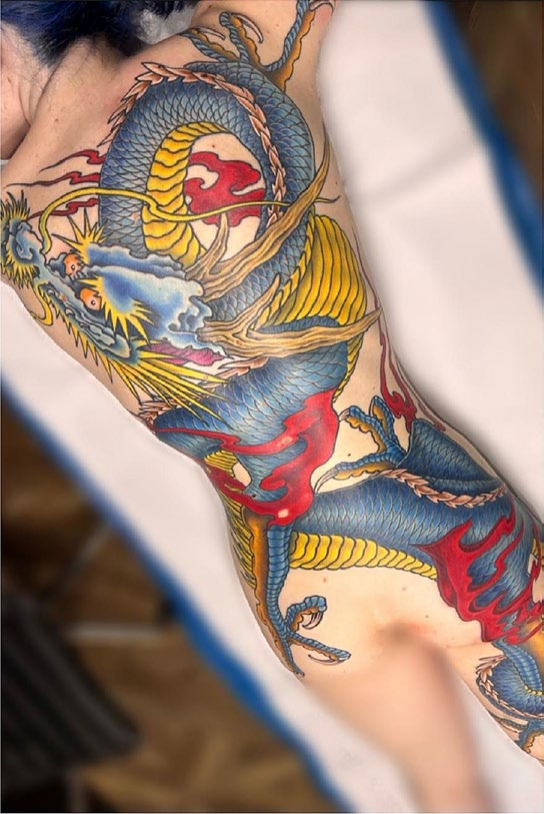 *Image provided by* steelpowertattoo, *with permission*	3
	Multicolor with pastels	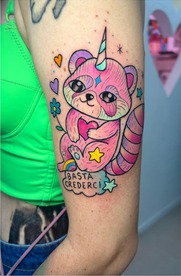 *Image provided by* amandatoy, *with permission*	5
Scarring	Present	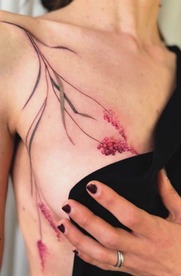 *Image provided by* gilbertavita, *with permission*	3
	Absent	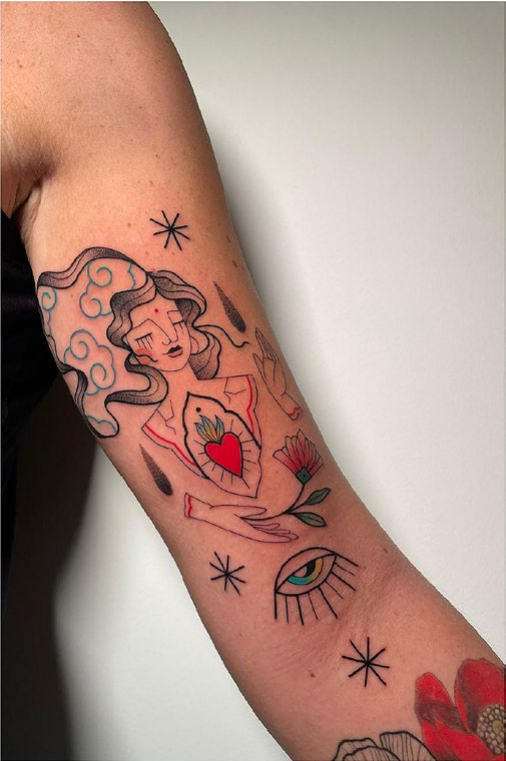 *Image provided by* lucille_roots, *with permission*	0
Layering (Cover‐up)	Present	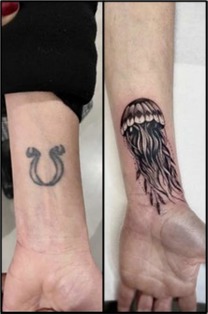 *Image provided by* vale.bonelli, *with permission*	2
	Absent	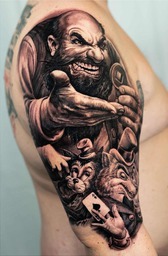 *Image provided by* perciastattoo, *with permission*	
Amateur tattoo			−2

Pigment density is assessed through a standardized clinical visual evaluation performed on baseline photographs and direct clinical examination prior to treatment. Density is classified as low (1 point), medium (2 points), or high (3 points) based on the overall visual saturation, opacity, and concentration of pigment within the tattooed area, taking into account uniformity of ink deposition and degree of dermal coverage.

Anatomical location is scored by assigning 2 points to areas known to respond poorly to laser treatment—hands, feet, and bony prominences—while all remaining sites receive 1 point. This distinction reflects well‐recognized differences in vascularization and lymphatic clearance.

Ink color composition is stratified into three categories: black or red monochrome (1 point), multicolor without pastels (3 points), and multicolor including pastel shades (5 points), with scoring based on the differential laser responsiveness and chemical composition of various pigments.

The presence of scarring or tissue changes within the tattoo area adds 3 points, while layered or cover‐up tattoos add 2 points, accounting for increased pigment density and treatment complexity.

The cumulative PT score provides a quantitative prediction of treatment sessions required, enabling standardized pre‐treatment counseling and individualized treatment planning.

### 2.2 Data Collection and Model Definition

Participants were eligible for inclusion if they were adults (≥ 18 years) and had undergone laser tattoo removal using the Discovery Pico laser (Quanta System, Italy) between 2019 and 2024 at the study centre. Subjects were excluded in the presence of active inflammatory dermatoses at the treatment site (e.g., eczema, psoriasis), concomitant skin or systemic conditions potentially affecting treatment outcomes, or incomplete clinical records. The final dataset comprised *n* = 545 participants, whose tattoos were treated using either single‐pass picosecond Nd:YAG (1064 nm) on full beam (fluence range: 1–4 J/cm^2^, spot size 4 mm, pulse duration 450 ps) or fractioned beam (0.45–0.7 J/cm^2^, spot size 8 mm), or full beam picosecond KTP (532 nm, fluence range: 0.45–0.7 J/cm^2^, spot size 4 mm, pulse duration 370 ps). All modalities were delivered at a repetition rate of 2 Hz. For each individual, the KD scale and the proposed PT scale were completed.

The target variable was the number of treatment sessions required for complete tattoo removal, defined as a discrete numeric value. The final dataset included 13 variables: six KD items, six PT items, and the target variable. No missing data were observed within the dataset. All items in the two scales were treated as categorical ordinal variables, as they represented point‐based scoring. All analyses were performed using Python 3.13.

Pairwise correlations among all variables were assessed using Spearman's rank correlation (*ρ*) [13]. Correlations were computed for each item of the KD and PT scales, for the total scores of both scales, and for the target variable (number of treatment sessions). A correlation matrix and corresponding heatmap were generated to support subsequent analysis.

The dataset was randomly divided into a training set (80%) and a test set (20%). Stratification in the split was applied to preserve the distribution of the target variable across both subsets, ensuring that the frequency of the outcome was consistent with the full dataset.

Normality was assessed for each variable in the training set using the Kolmogorov–Smirnov test [14]. As all variables exhibited non‐normal distributions, they were standardized by subtracting the median and dividing by the interquartile range. The training set was subsequently divided into two feature‐specific subsets: one containing only the KD scale variables (KD_train) and one containing only the PT scale variables (PT_train).

A 5‐fold cross‐validation grid search was performed separately on the KD_train and PT_train datasets to identify the optimal model and corresponding hyperparameters [15]. In each iteration, one fold served as the validation set, while the remaining four constituted the reduced training set. The number of folds was decided a priori to maintain an 80%–20% ratio between the training and validation sets, consistent with the one between training and test sets. Within the cross‐validation, eight regression models were evaluated:

- Linear
- Lasso
- Ridge
- Poisson
- Decision Tree
- Random Forest
- Multi‐Layer Perceptron
- Gradient Boost

For each model, hyperparameters were optimized through an exhaustive grid‐search over predefined parameter grids. The 5‐fold grid search resulted in one fitted model per fold for each algorithm. The best‐performing model, corresponding to the optimal hyperparameters, was identified based on the highest negative Root Mean Square Error (nRMSE) [16]. RMSE assigns greater weight to large prediction errors, with lower values indicating better performance. As the grid‐search follows a maximization criterion [17], the negative formulation of the RMSE was applied.

Following selection of the optimal model and corresponding hyperparameters, the final model was trained on the full training set and evaluated on the test set. Model performance was assessed using Mean Absolute Error (MAE) [18], that quantifies the average absolute deviation between predicted and observed values; the RMSE [16]; and the coefficient of determination (*R* ^2^) [19], that measures the proportion of variance in the target variable explained by the model.

Test set performance was compared across the optimal KD and PT models, as well as for the baseline models obtained by summing the item scores of each scale. Wilcoxon rank‐sum test [20] was applied to compare the RMSE distribution across the four models to evaluate statistical differences in predictive accuracy, using Holm‐Bonferroni correction to account for multiple tests comparison [21]. The best model was identified as the one resulting in the lowest MAE and RMSE and the highest *R* ^2^.

SHapley Additive exPlanations (SHAP) analysis was performed to quantify and interpret the contribution of each feature to the predictions of the best‐performing model [22]. SHAP values were calculated for all test set samples to quantify the magnitude and direction of each feature on individual predictions relative to the expected model output. Global feature importance was evaluated using two complementary visualizations: a SHAP summary plot, identifying the distribution of feature effects across all test observations; and a feature‐importance plot based on mean absolute SHAP values, providing a ranking of predictors according to their overall contribution.

Based on SHAP values, a recursive feature elimination was performed. At each iteration, the feature with the lowest cumulative importance was removed, and the model was retrained on the reduced training set. Performance was evaluated on the correspondingly reduced test set, and the optimal feature subset was defined as the set of predictors resulting in the lowest MAE and RMSE and the highest *R* ^2^.

## 3 Results

Baseline characteristics of the full cohort and the train/test subsets are presented in Table 2. The distribution of skin phototypes, KD scale items, and PT scale items was consistent across the training and test sets, with no statistically significant differences observed between subsets, confirming the representativeness of the stratified split.

**TABLE 2:** Distribution of skin phototype and tattoo characteristics according to the Kirby‐Desai and PT scales across the full cohort and the test and training subsets.

	Full cohort (*n* = 545)	Test subset (*n* = 109)	Training subset (*n* = 436)
Fitzpatrick Skin Type			
II	7 (1.3%)	1 (0.9%)	6 (1.4%)
III	454 (83.3%)	91 (83.5%)	363 (83.3%)
IV	78 (14.3%)	15 (13.8%)	63 (14.4%)
V	6 (1.1%)	2 (1.8%)	4 (0.9%)
Kirby‐Desai Scale | Tattoo Location			
Head and Neck	25 (4.6%)	4 (3.7%)	21 (4.8%)
Upper Trunk	218 (40%)	51 (46.8%)	167 (38.3%)
Lower trunk	83 (15.2%)	17 (15.6%)	66 (15.1%)
Proximal Extremity	156 (28.6%)	27 (24.8%)	129 (29.6%)
Distal Extremity	63 (11.6%)	10 (9.2%)	53 (12.2%)
Kirby‐Desai Scale | Tattoo Color			
Black only	428 (78.5%)	81 (74.3%)	347 (79.6%)
Black with some red	46 (8.4%)	12 (11%)	34 (7.8%)
Mostly black and red with other colors	40 (7.3%)	11 (10.1%)	29 (6.6%)
Multiple colors	31 (5.7%)	5 (4.6%)	26 (6%)
Kirby‐Desai Scale | Ink amount			
Amateur	25 (4.6%)	7 (6.4%)	18 (4.1%)
Minimal	274 (50.3%)	50 (45.9%)	224 (51.4%)
Moderate	216 (39.6%)	48 (44%)	168 (38.5%)
Significant	30 (5.5%)	4 (3.7%)	26 (6%)
Kirby‐Desai Scale | Scarring			
No	534 (98%)	108 (99.1%)	426 (97.7%)
Minimal	5 (0.9%)	1 (0.9%)	4 (0.9%)
Moderate	6 (1.1%)	0 (0%)	6 (1.4%)
PT Scale | Tattoo Type			
Realistic/Etching	87 (16%)	19 (17.4%)	68 (15.6%)
Geometric/Ornamental	155 (28.4%)	28 (25.7%)	127 (29.1%)
Old School/Blackwork	189 (34.7%)	40 (36.7%)	149 (34.2%)
Lettering/Script	96 (17.6%)	18 (16.5%)	78 (17.9%)
Tribal	18 (3.3%)	4 (3.7%)	14 (3.2%)
PT Scale | Pigment Density			
Low	25 (4.6%)	7 (6.4%)	18 (4.1%)
Medium	274 (50.3%)	50 (45.9%)	224 (51.4%)
High	246 (45.1)	52 (47.7%)	194 (44.5%)
PT Scale | Tattoo Location			
Rest of the body	482 (88.4%)	99 (90.8%)	383 (87.8%)
Hands/Feet/Bony prominences	63 (11.6%)	10 (9.2%)	53 (12.2%)
PT Scale | Tattoo Color			
Black or Red only	474 (87%)	93 (85.3%)	381 (87.4%)
Multicolor–no pastels	62 (11.4%)	16 (14.7%)	46 (10.5%)
Multicolor with pastels	9 (1.6%)	0 (0%)	9 (2.1%)
PT Scale | Scarring			
Absent	534 (98%)	108 (99.1%)	426 (97.7%)
Present	11 (2%)	1 (0.9%)	10 (2.3%)
Kirby‐Desai Scale and PT Scale | Layering			
Absent	513 (94.1%)	103 (94.5%)	410 (94%)
Present	32 (5.9%)	6 (5.5%)	26 (6%)
Session nr. (Mean ± SD)	8.4 ± 1.7	8.2 ± 1.5	8.4 ± 1.8

**FIGURE 1:**
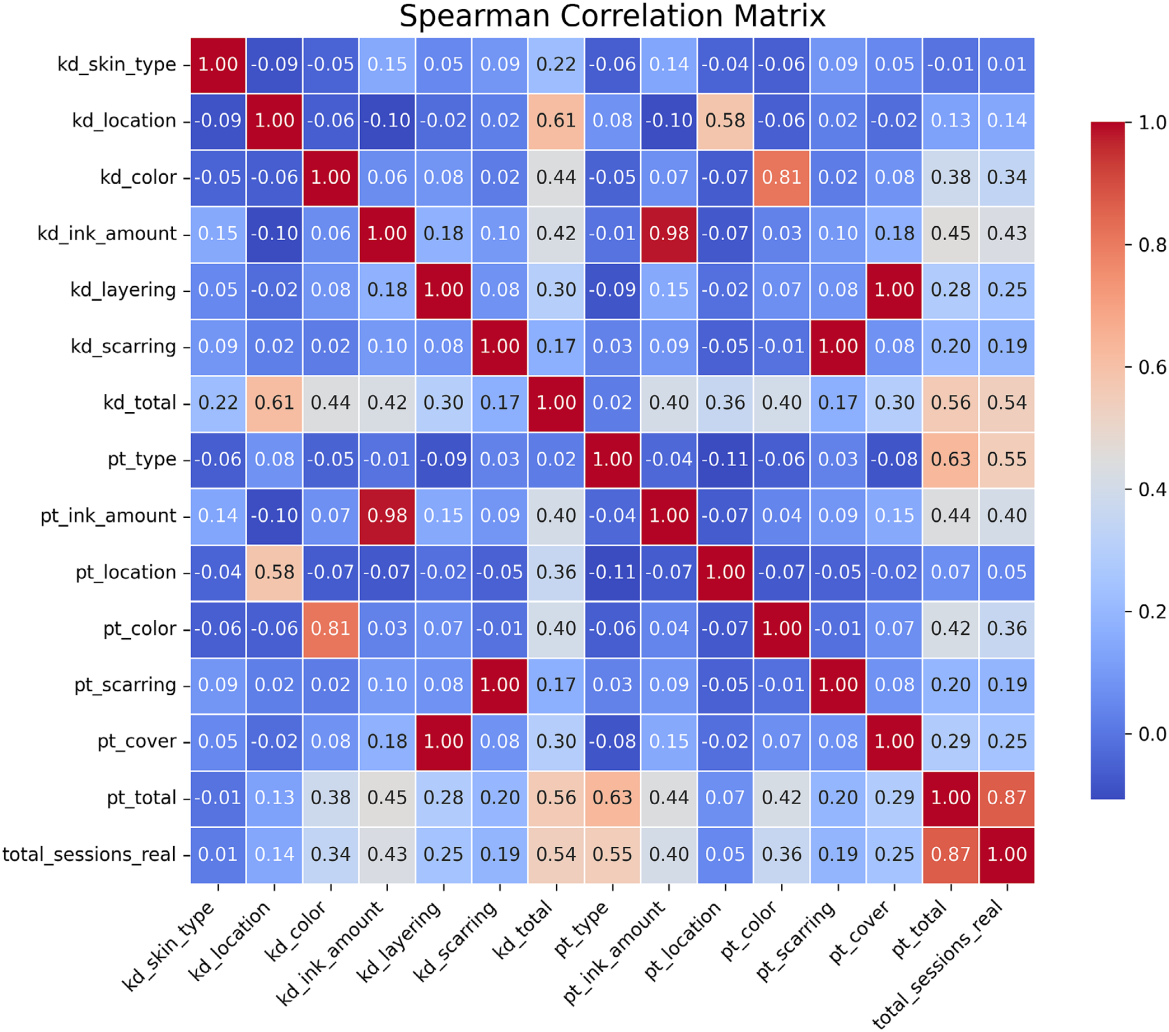
Correlation heatmap of the KD and PT scales. Scores are calculated using Spearman's *ρ*. Coefficients quantify the correlation between KD and PT scale items, their respective total scores (kd_total and pt_total), and the target variable (total_sessions_real).

In Figure 1, the correlation analysis is presented. Strong positive associations were observed among most items within and between the KD and PT scales. An exception was the KD “skin type” item, which was replaced in the PT scale by the “type of tattoo” item. These two variables showed no correlation with each other or with any other variable in the scales. A lower degree of association was observed between the two items related to tattoo coloration when compared to the other variables, indicating structural differences between the PT and KD scoring systems. The target variable (number of treatment sessions) did not correlate with any individual item of either scale. However, it exhibited a high correlation with the total PT score (*ρ* = 0.87) and a moderate correlation with the total KD score (*ρ* = 0.54).

In Table 3 the nRMSE scores for the regression models with the corresponding optimal hyperparameters are presented separately for the two datasets. The cross‐validation results for both scales showed limited variability across models, with nRMSE values ranging between −1.3293 and −1.4150 for the KD scale dataset and values ranging between −0.8248 and −0.9444 for the PT scale dataset. For the KD scale, Lasso regression achieved the best performance (nRMSE = −1.3293), slightly outperforming Linear and Ridge regressions. For the PT scale, Ridge regression provided the highest performance (nRMSE = −0.8248), followed by Lasso and Linear. For both datasets, non‐linear models such as Gradient Boost, Multi‐Layer Perceptron, and Random Forest yielded comparatively higher errors.

**TABLE 3:** Results of the models trained with optimal hyperparameters on the training set, reported separately for the KD dataset (left) and the PT dataset (right).

KD dataset model	nRMSE	PT dataset model	nRMSE
Linear	−1.330567	Linear	−0.826850
**Lasso**	**−1.329304**	Lasso	−0.824960
Ridge	−1.330445	**Ridge**	**−0.824771**
Poisson	−1.338990	Poisson	−0.842401
Decision Tree	−1.415019	Decision Tree	−0.944455
Random Forest	−1.351479	Random Forest	−0.895599
Multi Layer Perceptron	−1.351143	Multi Layer Perceptron	−0.830418
Gradient Boost	−1.336897	Gradient Boost	−0.861276

*Note:* Lower nRMSE values indicate better performance. The best‐performing models for each dataset are highlighted in bold.

Evaluation on the test set is presented in Table 4. It shows clear performance differences between models derived from the KD and PT scales. Among the KD‐based models, the simple sum score yielded the lowest overall accuracy, with the highest MAE and RMSE and a negative *R* ^2^. The KD Lasso model substantially improved performance and achieved a modest positive *R* ^2^, demonstrating limited but measurable predictive value.

**TABLE 4:** Results of the models trained with optimal hyperparameters on the test set.

Model	MAE	RMSE	*R* ^2^	*p* KD_sum	*p* KD_lasso	*p* PT_sum	*p* PT_Ridge
KD_sum	2.1376	2.4663	−1.5973	—			
KD_lasso	0.9958	1.3220	0.2537	< 0.001	—		
PT_sum	0.5046	0.9036	0.6513	< 0.001	0.0018	—	
PT_ridge	0.5804	0.6372	0.7280	< 0.001	0.0109	< 0.001	—

*Note:* For each model, the MAE, RMSE, and *R* ^2^ are reported, together with the *p*‐value resulting from the Holm–Bonferroni corrected Wilcoxon rank‐sum test for pairwise comparisons between models.

Models derived from the PT scale showed better overall performance. The PT sum score outperformed both KD‐based approaches. Although the PT Ridge model presented slightly higher MAE than the PT sum score, it achieved the lowest RMSE and the highest *R* ^2^ among all models, indicating smaller large‐error deviations and the highest proportion of explained variance. Overall, the PT Ridge model showed the best predictive performance on the test set.

The Wilcoxon rank‐sum test applied to the RMSE distributions produced statistically significant *p*‐values for all comparisons after Holm‐Bonferroni correction. These results indicate statistically significant differences in predictive performance across models and further support the superior accuracy of the PT Ridge model relative to the other approaches.

**FIGURE 2:**
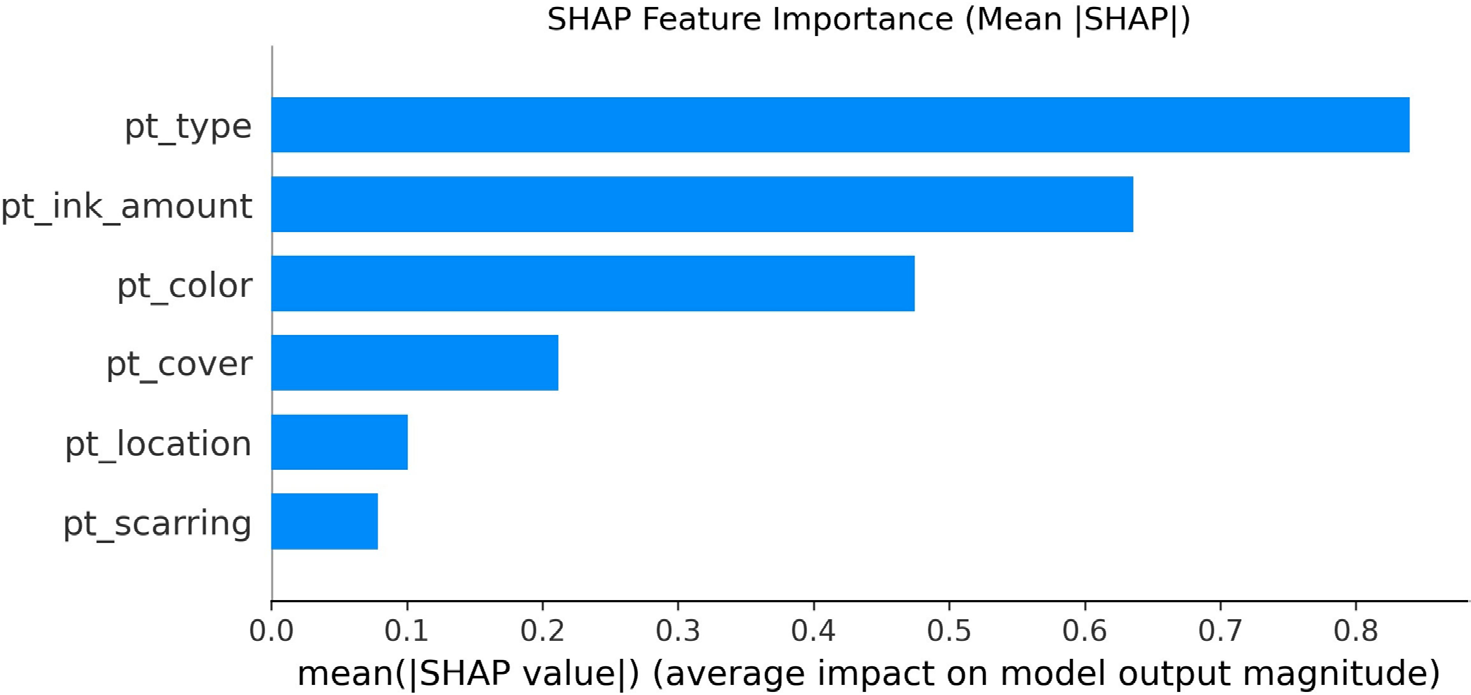
Mean absolute SHAP feature importance for the PT Ridge model. Bars represent the average contribution of each feature to the prediction of the number of treatment sessions.

In Figure 2, the global feature importance derived from SHAP values for the PT Ridge model is presented. The type of tattoo (pt_type) emerged as the most influential predictor, followed by the amount of ink (pt_ink_amount) and the color (pt_color) that showed large contributions to the output of the model. In comparison, covering, location, and scarring of the tattoo exhibited lower average SHAP magnitudes, indicating a more limited contribution to the prediction.

**FIGURE 3:**
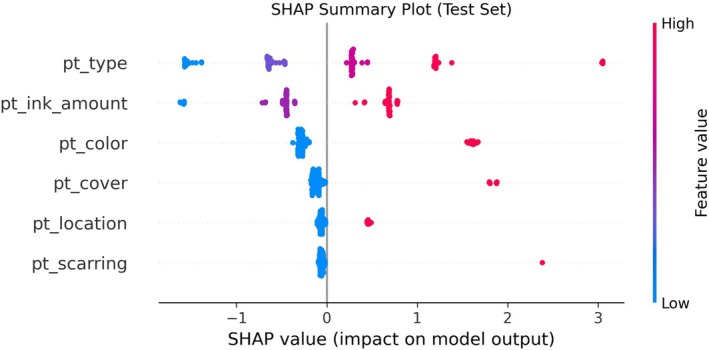
SHAP summary plot for the PT Ridge model on the test set. Each point represents the SHAP value for an individual observation, colored by the corresponding feature value.

Figure 3 provides a detailed summary of the feature effects across individual observations. For the type of tattoo and the amount of ink, both high and low values were associated with a wide range of SHAP values, confirming their strong influence on predicting the number of sessions. The other variables presented similar patterns, with higher values generally associated with increased predicted sessions. Tattoo location showed the narrowest distribution, indicating the least variable contribution.

Recursive feature elimination applied to the PT Ridge model did not result in improvements in MAE, RMSE, or *R* ^2^ at any iteration. Removal of features consistently led to equal or worse performance compared with the full model, suggesting that despite lower individual importance of some predictors, all PT features were necessary to correctly predict the number of sessions for tattoo removal.

## 4 Discussion

Laser tattoo removal using selective photothermolysis invariably requires multiple treatments, as widely documented in the literature. However, accurately predicting the number of sessions required for a given patient remains a significant clinical challenge. This predictive complexity is exacerbated when utilizing picosecond laser technology, whose more recent introduction and different mechanism of action compared to traditional Q‐switched lasers have necessitated a re‐evaluation of existing predictive models. The present study developed and validated a new predictive scale (PT) on a large sample of 545 subjects, systematically comparing it with the Kirby‐Desai (KD) scale, currently the most widely used reference in clinical practice.

The stability and robustness of the proposed PT Ridge model are supported by the application of gold standard techniques in data analysis and machine learning. The model selection relied on repeated cross‐validation with an exhaustive grid‐search to tune the hyperparameters, followed by evaluation on a held‐out test set, reducing the risk of overfitting. Furthermore, performance comparisons were based on non‐parametric tests with Holm‐Bonferroni correction, providing a conservative assessment of statistical differences among models. Finally, SHAP‐based interpretability and recursive feature elimination showed that, although some PT items had lower individual importance, removing them did not improve MAE, RMSE, or *R* ^2^, suggesting that the full set of PT features forms a stable representation of the number of days required to completely remove a tattoo. These elements indicate that the proposed model is more accurate than KD‐based approaches, and also methodologically sound and completely reproducible.

In recent studies, the Kirby–Desai (KD) scale has been shown to overestimate the number of treatment sessions required for complete tattoo removal with both nanosecond [10, 23] and picosecond laser systems [11]. This tendency is reflected in the high MAE (2.1376) and RMSE (2.4663) obtained by the KD sum model in the presented analysis. Conversely, the proposed scale, together with the PT Ridge model, demonstrates lower MAE and RMSE, and higher *R* ^2^, aligning with the reduced session counts consistently reported in current scientific literature. When compared with the only published data‐driven model, to the authors' knowledge, that refines KD predictions via multivariable regression for picosecond laser removal [24], that similarly reports KD overestimation, the PT Ridge approach is trained on a larger dataset, explicitly handles non‐normality in the ordinal predictors, and leverages modern machine learning and SHAP‐based interpretability methods, resulting in greater transparency, methodological robustness, and improved performances.

Our scale introduces the concept of “tattoo type” as a primary predictive parameter, with a differentiated scoring system (7, 5, 4, 3, 2 points) that reflects the technical complexity and pigment density characteristic of different artistic genres. This parameter represents an evolution from the binary amateur/professional classification of the KD scale, recognizing that modern tattoo art encompasses multiple styles with distinct physical–chemical characteristics that influence the outcome of laser treatment. The score is assigned based on technical complexity, pigment density, and color layering, directly reflecting the difficulty of removal. For example, realistic or massive tattoos, which require high pigment saturations and deep ink deposition to achieve three‐dimensional effects and complex shading, typically require more sessions than minimalist or geometric designs. This categorization is supported by the observations of Kirby et al., who found that pigment density in the dermis is a determining factor in the effectiveness of laser removal [4].

Our scale also recognizes the fundamental distinction between amateur and professional tattoos through a score adjustment system: when a tattoo is identified as amateur, 2 points are subtracted from the total accumulated score. This correction reflects the substantial differences in application technique—pigment deposited less uniformly, at varying depths, and often with a lower concentration—which generally makes the removal of an amateur tattoo faster than a professional equivalent.

The quantification of pigment density (high, medium, low: 3, 2, 1 points) is the central parameter of our predictive model. This approach is in line with a recent study by Menozzi‐Smarrito et al. [24], which has shown that ink density significantly influences the number of sessions required, regardless of the size of the tattoo. During laser tattoo removal, photons penetrate pigment aggregates, inducing their fragmentation into progressively smaller particles that are subsequently cleared by dermal macrophages and transported through the lymphatic system. This clearance process is more efficient for smaller, sub‐micrometric particles, whereas higher pigment density requires multiple sessions to gradually reduce the overall particulate burden to levels that can be effectively managed by the lymphatic system.

The PT scale simplifies anatomical categorization compared to the KD scale, which ranges from 1 (Head and Neck) to 5 (Distal Extremities). Our model distinguishes between problematic locations (hands, feet, bony prominences: 2 points) and standard locations (rest of the body: 1 point). This dichotomization is based on the differential distribution of vascularization and lymphatic drainage in different anatomical regions. As documented by Kirby et al., the head and neck have the highest concentration of regional lymph nodes and abundant vascularization, resulting in a more effective immune response in the phagocytosis of ink particles. The upper and lower trunk similarly maintain significant vascularization and lymphatic drainage. In contrast, the distal extremities, particularly the ankles, feet, hands, and fingers, have reduced lymphatic supply, compromising the efficiency of the pigment elimination process [4]. In their study, Menozzi‐Smarrito et al. confirmed that tattoos located on the ankles, feet, hands, and fingers systematically require a greater number of sessions than those on the trunk or neck [24]. Bony prominences are also anatomical sites where the reduced amount of subcutaneous tissue further limits lymphatic and vascular circulation.

The classification of colors in the PT scale (black/red: 1 point; multicolor: 3 points; multicolor with pastel colors: 5 points) reflects the complexity of the chemical composition of the pigments and their differential response to laser wavelengths. Black pigments, composed mainly of carbon and iron granules ranging in size from 0.5 to 4.0 μm, are the most easily removable type due to their ability to absorb all wavelengths of light. Red pigments, containing a mixture of metallic and carbonaceous elements with small percentages of titanium dioxide, also show good responsiveness to laser treatment. In contrast, pigments of other colors can reach sizes up to twice those of black pigments and contain varying proportions of metallic elements that complicate their removal. Pastel colors present the greatest challenge: typically obtained by dilution with white titanium dioxide, these pigments can paradoxically darken due to the photothermal effect during laser treatment, requiring more complex treatment protocols and a greater number of sessions.

Incorporating the “scarring” parameter into the PT scale acknowledges the substantial impact of dermal fibrosis on laser tattoo‐removal outcomes. This parameter accounts for both post‐tattoo scarring—arising from excessive fibroblast activation during the normal healing response following tattoo application—and pre‐existing traumatic scars subsequently camouflaged with tattoo ink.

In the first scenario, the physiological post‐tattoo healing cascade involves immune‐cell extravasation and stimulation of fibroblasts, macrophages, and neovascular tissue. When fibroblast activity becomes dysregulated, collagen overproduction occurs, ultimately leading to permanent dermal scarring. Similarly, when tattoo ink is deposited over pre‐existing traumatic scars, pigment becomes embedded within fibrotic dermal tissue that is significantly more resistant to laser treatment. This reduced responsiveness is attributable to two key mechanisms: (1) the dense collagen architecture, which attenuates laser energy penetration and diminishes the efficiency of photomechanical pigment fragmentation, and (2) impaired vascular and lymphatic networks, which significantly limit the clearance of fragmented pigment particles. For these reasons, the PT scale assigns a fixed value of three points to the presence of scarring—regardless of whether fibrosis developed before or after tattoo application—recognizing that dermal fibrosis consistently represents a major barrier to successful laser tattoo removal.

Layered or “cover‐up” tattoos (present: 2 points; absent: 0 points) represent a special category in which a new tattoo is superimposed on an older one with the intention of concealing it. Since tattoo ink is translucent, effectively concealing a pre‐existing tattoo requires darker shades and a generally larger design in the new tattoo. This layering necessarily leads to an increase in the overall density of pigment in the dermis, with ink particles present at different depths and potentially of varying chemical composition if done by different artists at different times. The removal of cover‐up tattoos is therefore invariably more complex and requires a greater number of sessions than single tattoos of comparable size.

The predictive power of the PT scale intentionally deviates from certain established parameters, most notably the patient's skin phototype, traditionally considered a critical variable in laser protocols. The analysis of our cohort, supported by the Spearman correlation matrix, revealed that the skin type exhibited one of the lowest correlation coefficients (*ρ*) with the total number of treatment sessions required. This low *ρ*‐value suggests a minimal linear predictive relationship between the Fitzpatrick Skin Type and the efficacy endpoint, which can be attributed to specific mechanisms of picosecond laser technology.

Tattoo pigments are composed of relatively large particles (nm to μm), whereas epidermal melanin is present in much smaller, uniformly distributed granules (melanosomes). The pronounced photomechanical effect generated by picosecond pulses is intrinsically more effective in fracturing the larger, aggregated pigment particles found in tattoos. Moreover, the ultra‐short pulse duration is significantly shorter than the thermal relaxation time of melanosomes and surrounding tissue. This temporal specificity maximizes the generation of a photoacoustic shockwave which mechanically fragments the pigment, preventing the diffusion of heat into adjacent tissues and protecting the melanin.

Our observations align with findings reported by Menozzi‐Smarrito et al., who also noted that skin type did not exert a major impact on laser tattoo treatment outcomes in their cohort. Specifically, Menozzi‐Smarrito et al. found that, even among patients with skin type IV, the tendency toward requiring more sessions compared to types I, II, and III was not statistically significant. It is important to acknowledge, however, that the authors did not include patients with phototypes V and VI [24].

This analysis is based on routinely collected clinical data, and as such it is subject to the inherent constraints of real‐world retrospective designs. One limitation, for instance, is the absence of patient‐related variables such as age and gender that were not collected and therefore could not be used as possible predictors in the model. Similarly, tattoo age has not been incorporated, despite evidence from Menozzi‐Smarrito et al. showing that older tattoos (> 10 years) require fewer sessions, likely due to natural fading and gradual pigment degradation [24]. This parameter was excluded due to inconsistent documentation in our retrospective dataset, as patients often could not accurately recall tattoo application dates.

Furthermore, although multiple regression models were evaluated, analysis focused on classical machine learning algorithms. More advanced architectures could leverage hybrid image‐based predictors that might further enhance predictive accuracy.

## Author Contributions

Valerio Pedrelli and Matteo Tretti Clementoni contributed substantially to the conception and design of the work. Emanuele Tauro and Enrico Gianluca Caiani contributed to the analytical framework and to the interpretation of the results. Valerio Pedrelli, Emanuele Tauro, Alessandra Zevini, and Riccardo Barini drafted the manuscript. Enrico Gianluca Caiani critically revised the manuscript for important intellectual content. All authors reviewed and approved the final version of the manuscript.

## Funding

The authors have nothing to report.

## Ethics Statement

The authors confirm that this study was conducted in accordance with the principles of the Declaration of Helsinki. Given the retrospective and non‐interventional nature of the study, based exclusively on routinely collected and fully anonymized clinical data, formal approval from an Institutional Review Board or Ethics Committee was not required, in accordance with local regulations and the guidelines of the *Journal of Cosmetic Dermatology*. All patients had provided written informed consent for laser treatment and for the anonymized use of their clinical data for research purposes.

## Conflicts of Interest

Alessandra Zevini and Riccardo Barini are employed at El.En. Group. All the authors declare that the research was conducted in the absence of any commercial or financial relationships that could be construed as a potential conflicts of interest.

## Data Availability

The data that support the findings of this study are available from the corresponding author upon reasonable request.
